# DNA damaging agents and p53 do not cause senescence in quiescent cells, while consecutive re-activation of mTOR is associated with conversion to senescence

**DOI:** 10.18632/aging.100265

**Published:** 2010-12-31

**Authors:** Olga V. Leontieva, Mikhail V. Blagosklonny

**Affiliations:** Department of Cell Stress Biology, Roswell Park Cancer Institute, BLSC, L3-312, Elm and Carlton Streets, Buffalo, NY, 14263, USA

**Keywords:** p53, DNA damage, senescence, quiescence, rapamycin, mTOR

## Abstract

When the cell cycle is arrested, growth-promoting pathways such as mTOR (Target of Rapamycin) drive cellular senescence, characterized by cellular hyper-activation, hypertrophy and permanent loss of the proliferative potential. While arresting cell cycle, p53 (under certain conditions) can inhibit the mTOR pathway. Senescence occurs when p53 fails to inhibit mTOR. Low concentrations of DNA-damaging drugs induce p53 at levels that do not inhibit mTOR, thus causing senescence. In quiescence caused by serum starvation, mTOR is deactivated. This predicts that induction of p53 will not cause senescence in such quiescent cells. Here we tested this prediction. In proliferating normal cells, etoposide caused senescence (cells could not resume proliferation after removal of etoposide). Serum starvation prevented induction of senescence, but not of p53, by etoposide. When etoposide was removed, such cells resumed proliferation upon addition of serum. Also, doxorubicin did not cause senescent morphology in the absence of serum. Re-addition of serum caused mTOR-dependent senescence in the presence of etoposide or doxorubicin. Also, serum-starvation prevented senescent morphology caused by nutlin-3a in MCF-7 and Mel-10 cells. We conclude that induction of p53 does not activate the senescence program in quiescent cells. In cells with induced p53, re-activation of mTOR by serum stimulation causes senescence, as an equivalent of cellular growth.

## INTRODUCTION

Serum growth factors (GF) activate the GF-sensing network, which turns on both cell cycle progression and the mTOR pathway, which in turn stimulates cellular growth in size [1-5]. While growing in size, cells progress through the cell cycle and divide. Thus, in proliferating cells, cellular growth is balanced with cell division.

In normal cells, serum withdrawal both arrests the cell cycle early in G1, also known as G0 and deactivates mTOR. Cells become quiescent: they neither grow in size nor progress through the cell cycle. In contrast, cellular senescence is characterized by cellular hypertrophy (large and flat cell morphology), hypersecretory phenotype, beta-Gal-staining and permanent loss of proliferative potential [6-8]. Cellular senescence is not caused by serum GF withdrawal, but by stresses and oncogenic/mitogenic hyper-stimulation [9-15]. While not inhibiting mTOR, these stimuli incite responses blocking cell cycle.

In theory, if the cell cycle is blocked, while serum continues to activate GF-sensing pathways, cells will senesce [16,17]. For example, p21 causes cell cycle arrest without inhibiting mTOR, and thus causes senescence. Deactivation of mTOR by rapamycin prevented p21-induced senescence, converting p21-induced arrest into quiescence [18-20].

The tumor suppressor p53 inhibits the mTOR pathway upstream [21-24] and downstream [25,26] of mTOR. While inhibiting mTOR, p53 suppressed p21-induced senescence, causing quiescence instead [27]. p53 affects autophagy and metabolic pathways not only via inhibition of mTOR but also probably independently from mTOR [22,28-35]. We use the term mTOR-centric network to encompass not only upstream and downstream but also parallel and TOR-like pathways [36].

p53 can both induce and suppress cellular senescence [37]. First, p53 causes cell cycle arrest, a prerequisite of senescence. Second, p53 inhibits mTOR-centric network and this can prevent senescence, causing quiescence instead. In cell lines with overactivated mTOR, p53 causes senescence [37]. Similarly, “weak” p53 that is not able to inhibit mTOR causes senescence simply by arresting the cell cycle [38]. In other words, p53 causes senescence passively by failing to suppress the senescence program (which in part depends on mTOR), while still causing cell cycle arrest. This model suggests that cell cycle arrest is the only mechanism of how p53 causes senescence. This predicts that induction of p53 will not cause senescence in quiescent cells, since in quiescent cells mTOR is already inhibited. Here we tested this hypothesis.

**Figure 1.**
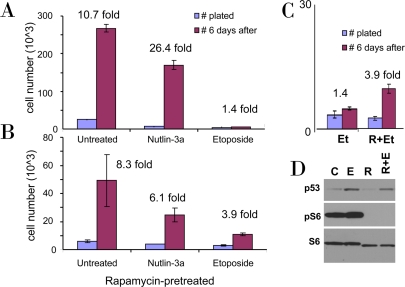
Rapamycin pretreatment prevents loss of proliferative potential during etoposide treatment. **A**-**C**. WI-38t cells were plated at 10000 cells/well in 24-well plates, and the next day were either left untreated (**A**) or pretreated with 10 nM Rapamycin (**B**). The next day, cells were treated with either 2.5 μM nutlin-3a or 1 μg/ml etoposide or left untreated. After 4 days, cells were trypsinized and 10% of cells were plated in fresh drug-free medium (blue bars). 6 days later cells were counted (red bars). In **C** the results for etoposide treatment (Et) with or without rapamycin (R) pretreatment are shown in the same scale. (**D**) Cells were lysed after 24 hr treatment with etoposide (E), rapamycin (R), or both (R+E) and immunoblot was performed.

## RESULTS

### Induction of p53 by etoposide in quiescent cells has little consequence

As we recently demonstrated, unlike nutlin-3a (an Mdm-2 antagonist), low concentrations of doxorubicin (DOX), a DNA damaging drug (DDD), caused senescent morphology in WI-38t cells [38]. Nutlin-3a causes cell cycle arrest solely by inducing p53, which in turn can inhibit the mTOR pathway. DOX causes cell cycle arrest at concentrations that induce p53 not high enough to inhibit mTOR. Therefore, DOX caused senescence as was determined by senescent morphology [38]. However, DOX is not washable and we could not check whether the condition was irreversible. Here we used etoposide, a DDD that could be washed out. We treated WI-38t cells with either etoposide or nutlin-3a. After 4 days, cells were washed and re-plated without drugs (Figure 1A). After 6 days, the number of nutlin-treated cells increased ~26 fold, whereas etoposide-treated cells could not proliferate (Figure 1A). In parallel, cells were treated with nutlin-3a and etoposide in the presence of rapamycin (Figure 1B). Rapamycin partially sustained the proliferative potential (PP) in etoposide-treated cells. Direct comparison of the proliferative potential of WI-38t cells treated with etoposide in the absence or presence of rapamycin is shown in Figure 1C. Etoposide did not inhibit mTOR and inhibition of mTOR by rapamycin-pretreatment (Figure 1D) favored quiescence over senescence (Figure 1C).

**Figure 2.**
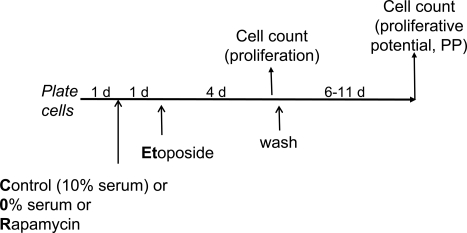
Experimental schema: transient induction of p53 in proliferating versus quiescent cells Cells are treated (or left untreated) under different condi-tions [control (10% serum), 0% serum or rapamycin] with etoposide for 4 days. Cells are counted twice: 1) at the time of etoposide removal to measure inhibition of proliferation and 2) 6-11 days after wash to measure proliferative potential (PP). PP should not be confused with proliferation. Thus, rapamycin and 0% serum inhibit proliferation but preserve (increase) proliferative potential in etoposide-treated cells.

**Figure 3.**
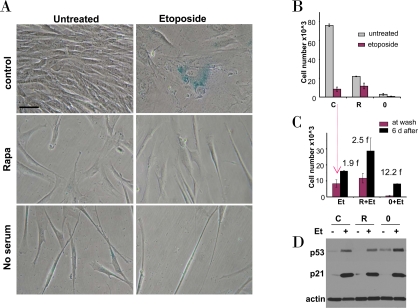
Effects of rapamycin and serum starvation on etoposide-induced senescence in WI-38t cells. **A-C.** WI-38t cells were plated at 5000/well in 12 well plates, and the next day either treated with 10 nM rapamycin in complete medium (R), or placed in serum-free medium (no serum or 0), or left in complete medium (control). The next day, 1 μg/ml etoposide (Et) was added, as indicated. **A.** After 4 days, cells were stained for beta-Gal and microphotographed (bar - 50 micron). **B.** After 4 days, cells were counted: control (C), rapamycin (R), no serum (0). **C.** Proliferative potential. In replicate plates, cells were washed and incubated in complete, drug-free medium for 6 days and then counted (black bars). Note: red bars correspond to red bars in panel B. Fold (f) increase in a cell number after drug removal. **D.** Immunoblot. Cells were plated in 6 well plates. The next day, cells were treated with 1 μg/ml etoposide (Et) for 24 hrs: control -C, rapamycin - R, no serum −0.

We further investigated effects of etoposide in cells treated by either rapamycin or serum starvation as depicted in Figure 2. Exposure of WI-38t cells to either rapamycin or serum starvation resulted in a lean cellular morphology, a characteristic of quiescence (Figure 3A, left column). Treatment of proliferating WI-38t cells with etoposide caused senescent morphology (Figure 3A, top right panel). Senescent morphology was partially preventable by rapamycin and serum-starvation: most cells were lean and thin (Figure 3A, right column). Rapamycin did not inhibit proliferation completely but rather slowed it down (Figure 3B). In agreement with similar experiment (Figure 1C), rapamycin partially prevented loss of proliferative potential caused by etoposide (Figure 3C). Serum starvation preserved proliferative potential (PP) in etoposide-treated cells (Figure 3C). Etoposide induced p53 in serum-starved cells even stronger than in control (proliferating) cells (Figure 3D). So the failure to initiate senescence could not be explained by lack of p53 induction.

**Figure 4.**
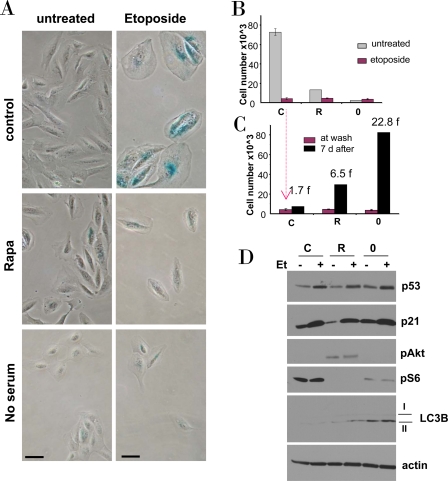
Effects of rapamycin and serum starvation on etoposide-induced senescence in RPE cells. **A-C.** RPE cells were plated at 5000/well in 12 well plates, and the next day either treated with 10 nM rapamycin in complete medium (R), or placed in serum-free medium (no serum or 0), or left in complete medium (control). The next day, 0.5 μg/ml etoposide (Et) was added, as indicated. **B.** After 4 days, cells were stained for beta-Gal and micro-photographed (bar - 50 micron) **C.** Proliferative potential. In replicate plates, cells were washed and incubated in complete, drug-free medium for 6 days and then counted (black bars). Note: red bars corre-spond to red bars in panel B. Fold (f) increase in a cell number after drug removal. **D.** Immunoblot. Cells were plated in 6 well plates. The next day, cells were treated with 0.5 μg/ml etoposide (Et) for 24 hrs: control -C, rapamycin -R, no serum −0.

We next investigated etoposide-induced senescence in normal retinal pigment epithelial (RPE) cells. Etoposide caused senescent morphology in RPE cells (Figure 4A). Pretreatment with rapamycin and serum-starvation partially prevented senescent morphology caused by etoposide. The senescent morphology was associated with permanent loss of proliferative potential: cells could not resume proliferation, when etoposide was removed (Figure 4B, C). In rapamycin-pretreated cells and, especially, in serum-starved cells, etoposide-induced arrest was partially reversible. Both rapamycin and serum starvation inactivated the mTOR pathway, as measured by a decrease of S6 phosphorylation (Figure 4D), but did not prevent p53 and p21 induction by etoposide (Figure 4D). Noteworthy, rapamycin activated Akt (Figure 4D). Serum-starvation was more effective than rapamycin in preventing etoposide-induced senescence. This suggests that mTOR pathway is not the only pro-senescent pathway and that is why rapamycin was less effective than serum-starvation in preventing senescence. As an example, compared with rapamycin, serum starvation was a more potent inducer of autophagy as judged by accumulation of LC3B-II (Figure 4D).

### Effects of higher concentrations of etoposide

**Figure 5.**
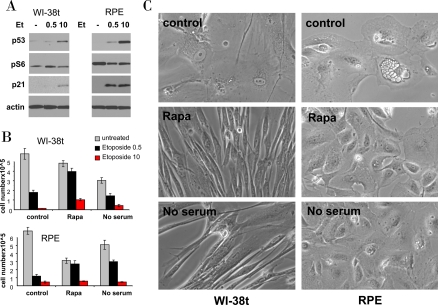
Effects of rapamycin and serum starvation on senescence caused by a higher concentration of etoposide. **A.** Immunoblot: WI-38t and RPE cells were treated with 0.5 μg/ml and 10 μg/ml etoposide (Et) or left untreated (-). The next day, cells were lysed and immunoblot was performed. **B-C:** WI-38t and RPE cells were plated at 25000/well in 12 well plates, the next day cells were either pretreated with 10 nM rapamycin (Rapa), placed in serum free medium (no serum) or left in complete medium with 10% serum (control). The next day, 0.5 μg/ml and 10 μg/ml etoposide (Et) was added: in complete medium (control) or with 10 nM Rapamycin (Rapa) or in serum free medium (no serum). After 5 days, cells were washed and cultured in fresh, drug free medium for 11 days and then trypsinized and counted. (in panel C): Before trypsinization, cells treated with 10 μg/ml etoposide (under three conditions: control, Rapa and no serum) were microphotographed.

We next investigated whether higher etoposide concentrations and durations of treatment convert quiescence into senescence. Like 0.5 μg/ml, 10 μg/ml etoposide did not inhibit mTOR (Figure 5A) and thus caused senescent morphology and loss of proliferative potential (Figure 5B, C). In WI-38t cells, rapamycin pretreatment partially preserved proliferative potential in both concentrations of etoposide (Figure 5B). Serum-starvation was insignificantly effective probably due to its toxicity during a 6-day treatment. At the time of cell count (Figure 5B), WI-38t cells treated with etoposide alone retained senescent morphology, whereas co-treatment with rapamycin and serum starvation (no serum) abrogated senescent morphology (Figure 5C). In RPE cells, which are more sensitive to etoposide, rapamycin and serum-starvation significantly preserved proliferative potential of cells treated with 0.5 μg/ml but not 10 μg/ml etoposide (Figure 5B). Still these co-treatments abolished senescent morphology otherwise caused by 10 μg/ml etoposide (Figure 5C).

### Conversion from quiescence to senescence

**Figure 6.**
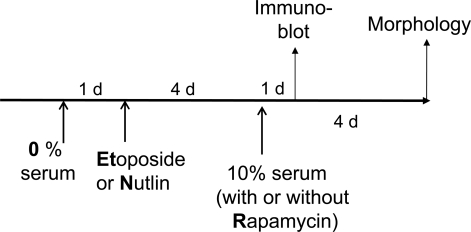
Schema. Serum stimulation of quiescent cells locked by p53.

**Figure 7.**
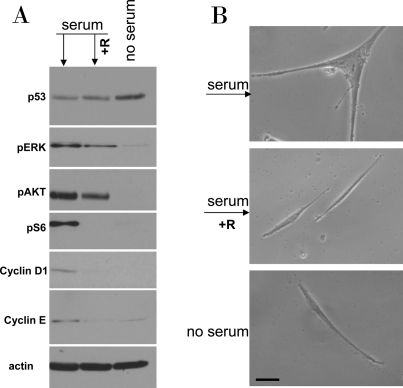
Serum stimulation of etoposide-locked WI-38t cells results in mTOR-dependent sensecence. **A-B**. WI38t cells were treated with 1μg/ml etoposide in the absence of serum as shown in Figure 6. Then, 10% serum was added either with 10 nM rapamycin (+R) or alone. No serum indicates that cells were continuously incubated with etoposide in serum free medium. 24 h after serum stimulation, cells were lysed and subjected to immuno-blotting as indicated (**A**). 4 days after serum stimulation cells were microphotographed (**B**).

**Figure 8.**
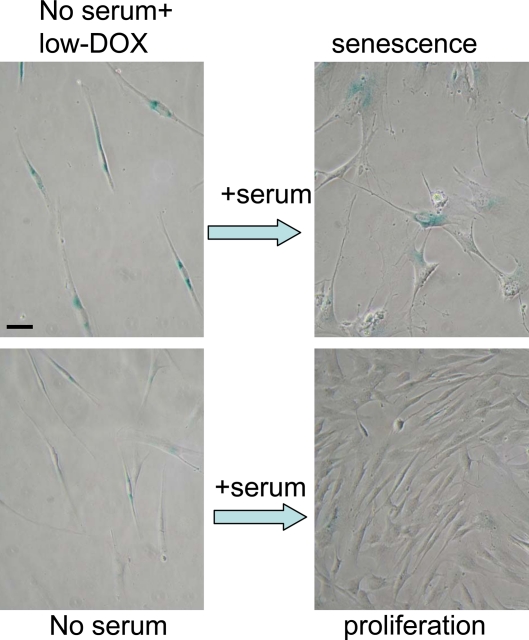
Serum stimulation converts Dox-locked quiescence into senescence in WI-38t cells. WI38t cells were treated with 100 ng/ml doxorubicin (low-Dox) or left untreated in serum-free medium (no serum) for 3 days, and then 10% serum was added. After 3 days of serum stimulation cells were stained for beta-gal and microphotographed. Bar - 50 micron.

**Figure 9.**
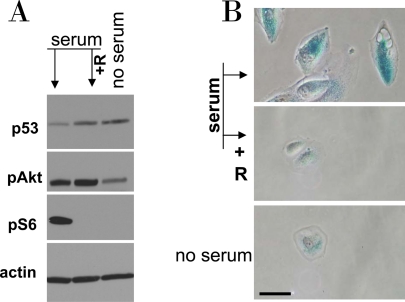
Serum stimulation of etoposide-locked RPE cells results in mTOR-dependent senescence. **A-B**. RPE cells were treated with 0.5 μg/ml etoposide in the absence of serum as shown in Figure 6. Then, 10% serum was added either with 10 nM rapamycin (+R) or alone. No serum indicates that cells were continuously incubated with etoposide in serum free medium. 24 h after serum stimulation, cells were lysed and subjected to immunoblotting as indicated (**A**). 4 days after serum stimulation cells were microphotographed (**B**).

**Figure 10.**
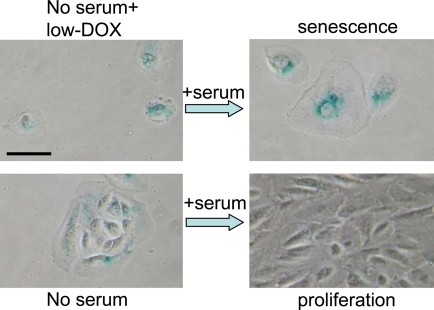
Serum stimulation converts Dox-locked quiescence into senescence in RPE cells. RPE cells were treated with 50 ng/ml doxorubicin (low-Dox) or left untreated in serum-free medium (no serum) for 3 days, then 10% serum was added. After 3 days of serum stimulation cells were stained for beta-Gal and microphotographed. Bar - 50 micron.

Using the schema depicted in Figure 6, we next investigated the effect of re-addition of serum to cells treated with DDD in serum-free medium without removal of the drug. In WI-38t cells, addition of serum caused phosphorylation of Akt, Erk and S6 and also induced cyclins D1 and E (Figure 7A). When stimulated with serum, these etoposide-arrested cells acquired senescent morphology (Figure 7B). Thus senescence was characterized by activated mTOR-centric pathways and elevated cyclins D1 and E. Rapamycin prevented S6 phosphorylation (downstream of mTOR), but not phosphorylation of Akt and Erk, which are upstream of mTOR. Simultaneously it diminished senescent morph-ology, so that most cells remained lean (Figure 7B). Similar results were obtained when cells were blocked with doxorubicin (Figure 8). Re-addition of serum caused senescence instead of proliferation (Figure 8). Similarly, in etoposide- or doxorubicin- blocked RPE cells, serum stimulation caused activation of mTOR (Figure 9A) and senescent morphology (Figure 9 and 10).

### Conversion between nutlin-induced quiescence and senescence in cancer cells

**Figure 11.**
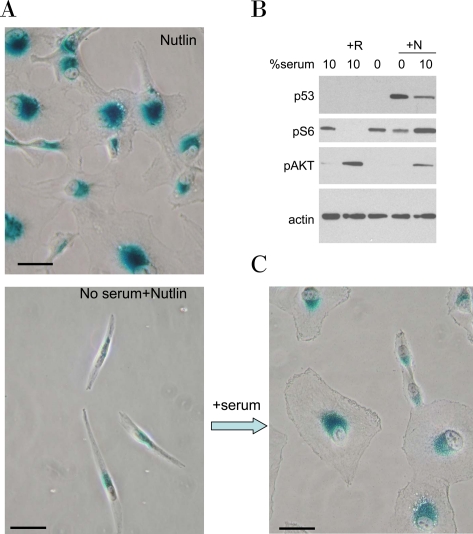
Serum stimulation is required for senescent morphology in nutlin-treated MEL-10 cells. **A.** MEL-10 cells treated with 2.5 μM nutlin-3a in the presence (Nutlin) or absence (No serum+Nutlin) of serum for 3 days were stained for beta-Gal and microphotographed (left panels). In parallel, 10% serum was added to a replicate well. After 3 days of serum stimulation cells were stained for beta-Gal and microphotographed (right lower panel). Bars - 50 micron. **B.** MEL-10 cells treated with 2.5 μM Nutlin-3a (N) in the absence or presence of 10% serum for 24 hr were lysed and subjected to immunoblotting, as indicated. Treatment with 10 nM rapamycin (R) is used as a control for mTOR inhibition.

**Figure 12.**
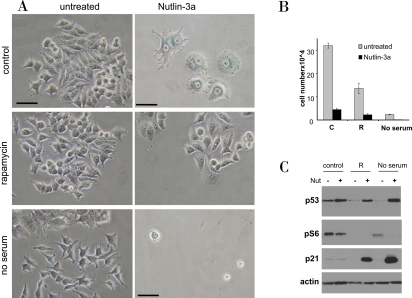
Rapamycin and serum starvation prevents nutlin-induced senescence in MCF-7 cells. **A-B**. MCF7 cells were plated at 5000 or 10000/well in 12 well plates, allowed to attach and then were either pretreated with 500 nM rapamycin (R), placed in serum free medium or left untreated in complete medium (control). The next day, 5μM nutlin-3a was added. After 5 days, cells were stained for beta-Gal and microphotographed (bars −50 micron) (**A**). (**B**) In replicate plate, cells were counted. **C.** MCF7 cells were treated as indicated for 24 hr, and immunoblot was performed.

Whereas nutlin-3a causes quiescence in WI-38t and RPE cells, it causes senescence in some cancer cell lines with high mTOR activity. For example, nutlin-3a did not block phosphorylation of S6 and caused senescence in Mel-10 cells. As we have shown, senescence was preventable by rapamycin [37]. The advantage of the nutlin-based model is that nutlin-3a does not cause DNA damage. Although serum-starvation did not cause genuine quiescence in cancer cells, serum starvation still prevented typical senescent morphology during treatment with nutlin-3a. Mel-10 cells remained slim, when were treated with nutlin-3a in serum-free medium. The cells were beta-gal positive (Figure 11A), because serum starvation alone may cause beta-gal staining. Serum-starvation by itself did not decrease p-S6 by day 1 (Figure 11B), there was a noticeable decrease in phosphorylation of S6 in nutlin-treated cells maintained in a serum-free medium (Figure 11B). Re-addition of serum converted lean morphology into typical senescent phenotype (Figure 11C). In MCF-7 cells, nutlin-3a also induced senescent morphology (Figure 12A) and in agreement did not inhibit S6 phosphorylation (Figure 12C). However, non-proliferating senescent cells co-existed with still proliferating cells, which formed colonies with non-senescent morphology. (Notably, higher concentration of nutlin-3a caused cell death, data not shown). Serum starvation slowed down proliferation of MCF-7 cells (Figure. 12B). Induction of p53 in serum-starved cells by nulin-3a caused rapid and massive cell death (Figure 12A, lower panel). Nutlin-3a induced especially high levels of p53 in serum-starved cells (Fig 12C). This can explain both inhibition of p-S6 and cell death, according to the recent model [38].

We also utilized rapamycin, which abrogated S6 phosphorylation, while did not prevent p53 induction by nutlin-3a (Figure 12C). In rapamycin-pretreated MCF-7 cells, nutlin-3a did not cause morphological senescence (Figure 12A). In other words, senescence was converted into quiescence. Unlike senescent cells, quiescent cells were not morphologically distinct from proliferating cells. So after treatment with nutlin-3a plus rapamycin, there could be a mixture of proliferating and quiescent cells. Therefore, in order to link the morphology to the proliferative potential of arrested cells, we needed to selectively eliminate proliferating cells first. This task unexpectedly merged with our investigation of drug combinations that could protect cells with wt p53 from the toxicity of chemotherapy. Using this approach, we demonstrated that rapamycin converted nutlin-induced senescence into quiescence in MCF-7 cells (MS in preparation).

## DISCUSSION

In this study we tested the idea that while causing senescence in proliferating cells, DNA damaging drugs and induced p53 will not cause senescence in quiescent cells. To this point we 1) induced quiescence prior to p53 induction and 2) used p53-inducing agents that could be washed out to observe whether treated cells would retain proliferative potential. Etoposide, which causes DNA damage, was used for normal cells and nutlin-3a, which induces p53 without DNA damage, was used for cancer cells. Both agents could be washed out to check the reversibility of arrest. We used these drugs at concentrations that caused senescence in proliferating cells. When applied to serum-starved and rapamycin-treated cells, p53-inducing drugs did not completely convert quiescence into senescence. Cells retained mostly quiescent morphology and some degree of proliferative potential, resuming proliferation in fresh (drug-free, serum-containing) medium.

Although not causing senescence, induction of p53 in quiescent cells ‘locked’ the cell cycle. In quiescence caused by serum starvation, the cell cycle is inactive (due to low levels of cyclins) but not blocked. In quiescent cells, induction of p53 blocks the cell cycle (in addition to its deactivation). Re-addition of serum to such blocked (locked) quiescent cells did not cause proliferation. Instead it caused senescence. This is in agreement with the notion that senescence is a form of growth, a continuation of growth, when proliferation is impossible [18,39].

Thus, induction of p53 by different agents (including DNA damaging drugs) did not cause senescence in serum-starved and rapamycin-treated cells. It is less clear whether DNA damaging agents induced identical DNA damage in all conditions. One potential problem is that DNA damaging drugs may induce a lesser DNA damage in quiescent cells. However, first, we utilized etoposide, which was reported to induce damage in all phases of the cell cycle [40-45]. Second, etoposide induced the same levels of p53 in proliferating, serum-starved and rapamycin-treated cells. (Note: Although p53 could be induced independently from DNA damage, this has not been described for etoposide. DNA damage response such as gamma-H2AX is also not absolutely reliable marker because it may occur in the absence of DNA damage in senescent cells [46,47]). Therefore, direct measurement of DNA damage by comet assay is warranted. 0.5 μg/ml etoposide did not induce obvious comets both in control and serum-free conditions. 10 μg/ml etoposide induced comets in serum-starved cells (Supplemental Figure 1). We conclude that, in agreement with literature data, etoposide induces DNA damage in serum-starved cells (some of which may still cycling at the moment of etoposide treatment) but more detailed measurements are needed for quantitative results. Third, simultaneous serum-withdrawal and addition of doxorubicin also suppressed senescence and this effect cannot be explained by cell cycle arrest caused by serum withdrawal. Fourth, DNA damaging drugs were even more cytotoxic in serum free-medium, indicating damage. Thus, it is not that etoposide is less cytotoxic in serum-free medium but rather that cells retain lean morphology and prolifertative potential (the ability to proliferate in fresh medium). At high concentrations, damaging agents and p53 can induce cell death rather than senescence in serum-starved cells. Yet, according to our preliminary data, if drugs are removed before death occurs, serum-restimulated survived cells can become senescent. TOR-independent latent senescence caused by high levels of DNA damage is an intriguing topic for further investigations.

In conclusion, quiescence is characterized by inactive mTOR both in cell culture [18,19] and in the organism [48,49]. The inability of p53 to cause senescence in quiescent cells has important physiological applications. Most cells of an adult organism are resting and there-fore induction of p53 and DNA damage cannot cause senescence. In contrast, stimulation of GF-sensing mTOR-centric pathways can. ‘Locked’ quiescent cells represent post-mitotic cells in the organism, including muscle cells, adipocytes and neurons. While not triggering proliferation of such ‘locked’ cells, stimulation with growth factors, hormones and nutrients may cause their senescence. Conversion of quiescence to senescence is a model of physiological senescence. Locked (non-senes-cent) cells undergo chronic over-stimulation and event-ually senesce. At least in some in vitro cellular models, conversion of quiescence to senescence (physiological senescence) can be suppressed by rapamycin.

## MATERIALS AND METHODS

### Cell lines and reagents.

WI-38-Tert, WI-38 fibroblasts immortalized, and RPE, retinal pigment epithelial cells were described previously [18]. RPE cells were cultured in MEM with 10% FBS, WI-38-tert cells were cultured in low glucose DMEM with 10% FBS. MEL-10, melanoma cell line, and MCF-7, breast cancer cell line, were cultured in DMEM (plus pyruvate) with 10% FBS. Rapamycin was obtained from LC Laboratories, MA, USA. Nutlin-3a, etoposide and doxorubicin were from Sigma-Aldrich.

### Immunoblot analysis.

Whole cell lysates were prepared using boiling lysis buffer (1%SDS, 10 mM Tris.HCl, pH 74.). Equal amounts of proteins were separated on 10% or gradient polyacrylamide gels and transferred to nitrocellulose membranes. The following antibodies were used: mouse anti-p53 (Ab-6) from Oncogene, mouse anti-p21 from BD Biosciences; rabbit anti-actin from Sigma-Aldrich; rabbit anti-phospho-S6 (Ser235/ 236), mouse anti-S6, rabbit anti-phospho AKT, rabbit anti-LC3B, anti-phospho ERK from Cell Signaling; anti-cyclins D1 and E from Santa Cruz Biotechnology. Secondary goat anti-rabbit and goat anti-mouse HRP conjugated antibodies were from Chemicon and Bio-Rad, respectively. Signals were visualized using ECL chemilumenescence kit from Pierce.

### SA-β-Gal staining.

Beta-Gal staining was performed using Senescence-galactosidase staining kit (Cell Signaling Technology) according to manufacturer's protocol. Cells were incubated at 37°C until beta-gal staining becomes visible. Development of color was detected under light microscope.

Neutral comet assay was performed according to manufacturer's protocol.

Proliferative potential was determined as described in detail in Figure legends.

## SUPPLEMENTAL FIGURES

Supplementary Figure 1. Comet assay RPE cells were seeded at 25,000 per well in 12-well plates. The next day, the medium was changed to 0% serum for 24 hours and then the cells were treated with 10 μg/ml etoposide for 1 hour and neutral comet assay was performed.
