# Supplementary figures and images for: DNA damaging agents and p53 do not cause senescence in quiescent cells, while consecutive re-activation of mTOR is associated with conversion to senescence
Source: Aging (Albany NY). 2010 Dec 31;2(12):924–35. doi: 10.18632/aging.100265 (PMC3034181; doi:10.18632/aging.100265)

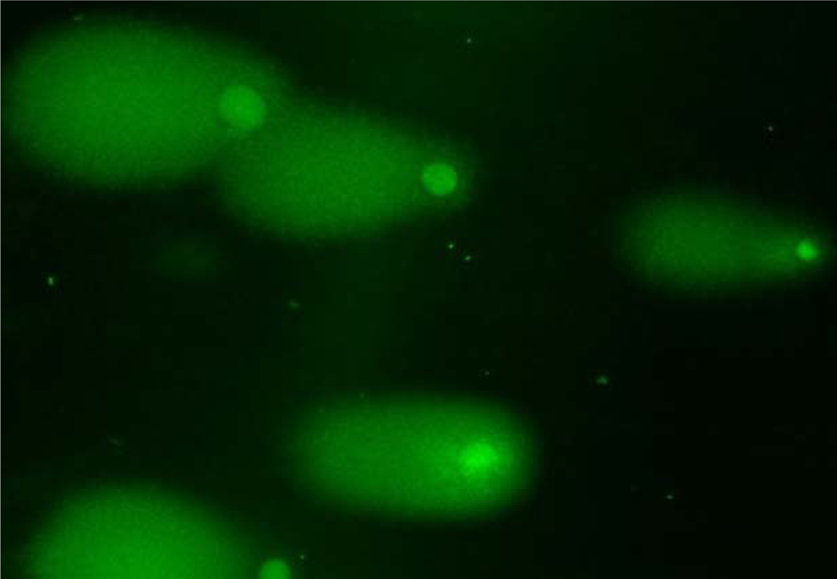

Supplement: Supplementary Figure 1. — RPE cells were seeded at 25,000 per well in 12-well plates. The next day, the medium was changed to 0% serum for 24 hours and then the cells were treated with 10 μg/ml etoposide for 1 hour and neutral comet assay was performed. [file aging-02-924-s001.tif]
